# Molecular Detection and Characterization of *Ehrlichia canis* Isolates from Three Geographic Regions in Mexico: A Retrospective Study

**DOI:** 10.3390/life13081629

**Published:** 2023-07-27

**Authors:** José Juan Lira-Amaya, Diana M. Beristain-Ruiz, Jesús Racanco-Delgado, Javier A. Garza-Hernández, Cuauhcihuatl Vital-García, Montserrat Santamaria-Espinosa, Grecia Martínez-García, Antonio Alvarez-Martínez, Andrés Quezada-Casasola, Carmen Rojas-Martínez, Beatriz Alvarado-Robles, Julio V. Figueroa-Millán

**Affiliations:** 1 CENID-Salud Animal e Inocuidad, INIFAP, Carretera Cuernavaca-Cuautla No. 8534, Jiutepec 62550, Morelos, Mexico; lira.juan@inifap.gob.mx (J.J.L.-A.); santamaria.rebeca@inifap.gob.mx (M.S.-E.); martinez.grecia@inifap.gob.mx (G.M.-G.); alvarez.jesus@inifap.gob.mx (A.A.-M.); rojas.carmen@inifap.gob.mx (C.R.-M.); 2 Departamento de Ciencias Veterinarias, Universidad Autónoma de Ciudad Juárez, Anillo Envolvente y Estocolmo s/n Colonia Progresista AP 1729-D Cd., Ciudad Juárez 32310, Chihuahua, Mexico; diana.beristain@uacj.mx (D.M.B.-R.); cuauhcihualt.vital@uacj.mx (C.V.-G.); aquezada@uacj.mx (A.Q.-C.); balvarad@uacj.mx (B.A.-R.); 3 Unidad Académica de Medicina Veterinaria y Zootecnia, Universidad Autónoma de Guerrero, Km. 3.5, de la Carretera Altamirano-Iguala, Ciudad Altamirano 39640, Guerrero, Mexico; racanco@hotmail.com; 4 Departamento de Ciencias Químico-Biológicas, Universidad Autónoma de Ciudad Juárez, Anillo Envolvente y Estocolmo s/n Colonia Progresista, Ciudad Juárez 32310, Chihuahua, Mexico; javier.garza@uacj.mx

**Keywords:** canine monocytic ehrlichiosis (CME), *Rhipicephalus sanguineus*, infection rates, serological assay, PCR assay, 16S rRNA gene

## Abstract

Canine monocytic ehrlichiosis (CME) is the most common tick-borne disease affecting domestic dogs and other wild canids. It has a worldwide distribution and is associated with the presence of the brown dog tick. Few studies have been conducted in Mexico to identify and characterize *Ehrlichia canis* genetic variability. In the present study, 111 dogs of different sex, breed, and age from three geographic regions in Mexico were included. All of them had a previous history of tick infestation and/or the presence of one or more clinical signs compatible with CME. All dogs were tested by a commercial ELISA and nested PCR assay for the detection of *E. canis.* In addition, we analyzed the *E. canis* genetic diversity from the 16S rRNA gene sequences obtained in this study, along with 15 additional sequences described for *E. canis* in Mexico and obtained from GeneBank. Serological detection by commercial ELISA results showed overall infection rates of 85.58% (95/111), including 73.1% (30/41) in samples from Guerrero state; 75% (15/20) in Morelos; and 100% (50/50) in Chihuahua. On the other hand, molecular detection (nPCR assay) showed 31.5% (35/111) overall infection rate, with 41.4% (17/41) in Guerrero state; 55% (11/20) in Morelos; and 14% (7/50) in Chihuahua. We observed a high 16S rRNA gene sequence conservancy in most of the *E. canis* isolates in the three geographical areas from Mexico, including those analyzed in this research, suggesting a common geographic origin among isolates.

## 1. Introduction

Ticks are hematophagous ectoparasites that can transmit a wide variety of pathogens such as viruses, bacteria, rickettsias, and protozoa to a wide range of hosts, including humans and animals [1]. Canine monocytic ehrlichiosis (CME), or tropical pancytopenia (TP), is one of the most common diseases in domestic dogs; the infection is transmitted by ticks and is caused by *Ehrlichia canis*, an obligate Gram-negative, intracellular rickettsia, that infects canine monocytes [2].

CME is endemic in tropical and subtropical areas around the world. Its presence is associated with the brown dog tick vector *Rhipicephalus sanguineus* [3]. This disease can present three clinical phases, acute, subclinical, and chronical [4,5]. There are many clinical signs associated to CME such as depression, lethargy, anorexia, weight loss, and hemorrhagic tendencies. Physical examination also reveals lymphoadenomegaly and splenomegaly [5]. It is a disease of global importance in small animal practice [6]; therefore, various methods have been developed for diagnostics, including molecular and serological techniques. Molecular techniques are based on detection and amplification of the pathogen DNA; they are useful to confirm the *E. canis* infection in acute cases. PCR assays are the most commonly used method to determine the molecular prevalence of the pathogen [5,6,7,8]. Whereas serological methods provide an overview of the immune status of animals, they demonstrate whether animals have previously been exposed to *E. canis* and have developed specific antibodies. For example, the Indirect Fluorescent Antibody Test (IFAT) and Enzyme-Linked Immunosorbent Assay (ELISA) are conventionally used in seroprevalence studies [5,7,8,9]. There are several previous investigations carried out to determine the prevalence of CME in the Americas. The molecular prevalence of *E. canis* in Central and South America ranges from 7% to 47.7%. For example, a molecular prevalence of 47.7% (148/310) has been reported in Costa Rica [10]; 25.6% (97/379) in Brazil [11]; whereas in Colombia [12], Paraguay [13], and Argentina [14], a 40.6% (37/91), 10.4% (40/384), and 7% (6/86) was reported, respectively. In Mexico, different studies report diverse molecular prevalence, such as 10% in La Laguna region [15]; between 29.2% (72/246) and 38.4% (92/246) for Yucatán state [16]; about 26.8% (103/384) in Tamaulipas state [17]; and up to 53.6% (104/194) in Paso del Norte region [18], the highest prevalence for *E. canis* so far reported. In most cases, dogs with CME usually respond favorably to treatment with doxycycline, while others develop the chronic phase, or even death in untreated dogs [6].

Although it is well known that *E. canis* has a worldwide distribution, few studies identify and characterize its genetic variability [19,20]. Further research is required to better understand the molecular and genetic diversity of *E. canis*, information that may notify on disease epidemiology and pathogenesis [20]. In general, *E. canis* 16S rRNA gene sequences are highly conserved among isolates from South America, North America, Asia, Europe, Africa, and the Middle East [20]. 

DNA sequencing and phylogenetic analysis of the gene encoding small subunit ribosomal RNA (16S rRNA gene) has been a useful tool for studying the evolution of *E. canis* and other bacteria [21,22]. A specific PCR assay that amplifies a segment of the 16S rRNA gene was proposed originally to identify different *Ehrlichia* species [23]. A highly variable region located near the 5’end of the 16S rRNA gene sequence [23] has been selected for a nested PCR (nPCR) assay in which primers HE and ECA define a 389-bp product upon PCR amplification [24]. Moreover, the 16S rRNA gene has previously been used as a target and the sequences obtained have been applied in the genetic characterization of *E. canis* strains in dogs from Peru [25], Greece [26], and, more recently, Turkey [27]. Previous *E. canis* studies in Mexico used 16S rRNA gene and trp36 as target genes for phylogenetic analysis [16,17,28,29,30]. 

The aim of this study was to estimate the frequency of *E. canis* infection rates in three different geographical regions of Mexico, and to identify the biogeographic relationships inferring their molecular phylogeny among 16S rRNA gene sequences priorly reported for Mexico.

## 2. Materials and Methods

### 2.1. Ethical Approval and Informed Consent

The study protocol was reviewed and approved by the Ethical and Bioethical Committee, Autonomous University of Juarez (CIBE-2016-1-16) Mexico. The study was performed in compliance with Mexican regulations on the use, housing, and transport of experimental animals (NOM-062-ZOO-1999 and NOM-051-ZOO-1995), and American guidelines for research on animals (Guide for the Care and Use of Laboratory Animals in National Resource Council, 2011).

### 2.2. Study Population

A cross-sectional descriptive study was conducted using non-probability sampling for convenience. The study population consisted of 111 purposively selected dogs referred between 2011 and 2015 to three small animal veterinary clinics in different geographic regions of Mexico: Ciudad Altamirano, Guerrero (*n* = 41), Ciudad Juárez, Chihuahua (*n* = 50) and Cuautla, Morelos (*n* = 20). Inclusion factors were one or more clinical signs consistent with canine monocytic ehrlichiosis (fever, anemia, lethargy, and epistaxis) or, the presence or recent history of tick exposure. 

### 2.3. Sample Collection 

Blood samples were collected by venipuncture from jugular or cephalic vein using vacuum tubes with EDTA as an anticoagulant. The components of the samples (packed cell volume and plasma) were separated by centrifugation at 3500 rpm for 5 min; plasma and packed cells (containing erythrocytes and leukocyte layer) were stored in the freezer at −20 °C until analyzed by ELISA and nPCR, respectively.

### 2.4. Serological Detection of E. canis Antibodies

The analysis of plasma samples was performed with the commercial ELISA (SNAP 4Dx Plus test; IDEXX Laboratories, Barcelona, Spain); each test was executed by following the manufacturer’s instructions.

### 2.5. DNA Extraction and Nested PCR Amplification of 16S rRNA Gene from E. canis

Extraction of genomic DNA was performed from the cell pack previously separated of samples using an Ultra Clean BloodSpin DNA Isolation commercial kit; MO BIO Laboratories (Carlsbad, CA, USA) by following the manufacturer’s instructions. Nested PCR involved two sequential amplification reactions. For each PCR round, specific oligonucleotides were used for amplification of the 5′ hypervariable region [23] of the 16S rRNA gene (Table 1) by using the PCR profile previously reported for *E. canis* [24]. Initial PCR reactions were prepared in a final volume of 25 μL; each reaction included 12.5 μL of GoTaq Green Master mix; PROMEGA (Madison, WI, USA), 5 μL of genomic DNA (30–50 ng), 1 μL of each oligonucleotide (10 pmol), and 5.5 μL of nuclease-free water. The nested PCR assay included same quantities of the Master mix, except 2 uL amplification product of the first round PCR, and 8.5 uL of nuclease-free water were used. After amplification, DNA fragments were visualized and analyzed by electrophoresis in 3% agarose gels stained with ethidium bromide. 

### 2.6. DNA Sequencing

DNA fragments (approximately 389 bp) of some positive samples obtained by nested PCR were purified from the agarose gel using Wizard^®^ SV Gel and PCR Clean-Up System kit; PROMEGA (Madison, WI, USA). DNA fragments were cloned into a TOPO TA cloning vector, Invitrogen (Carlsbad, CA, USA), and were subsequently submitted to the Biotechnology Institute UNAM, Mexico, for sequencing by the Sanger method with fluorolabeled dideoxynucleotides as chain terminators. The DNA fragments from Guerrero isolates were directly sequenced without performing the cloning process. Nucleotide sequences were aligned using the BLASTn program (https://blast.ncbi.nlm.nih.gov/Blast.cgi, accessed on 31 July 2022) to determine the DNA percent identity by searching the GenBank sequence database. 

### 2.7. Selection of Representative Sequences of E. canis and Phylogenetic Analysis

A GeneBank search was conducted, and 15 additional *E. canis* 16S rRNA gene sequences deposited from Mexico were included in the phylogenetic analysis. Keywords used for search were: Mexico, isolate, 16S ribosomal RNA gene, ticks, blood, and human. Then, FASTA format sequences were aligned using CLUSTAL-W (default parameters) performed using MEGA program, version 11. For construction of the phylogenetic tree, associations and molecular evolution of *E. canis* sequences were inferred using the Bayesian algorithm. Briefly, the aligned *E. canis* sequence FASTA files were first converted into Nexus files in MEGA. Then, to generate the BEAST file, the BEAUti software in the BEAST package was used performing the general time reversible (GTR) as substitution model, the Gamma + Invariant sites as heterogeneity model. The tree prior shared by all tree models was computed by the Yule speciation model using a random starting tree, and the MCMC chain was run for 108 generations to ensure convergence. Subsequently, the BEAST file was processed to build the phylogenetic tree. Then, the tree was built using the TreeAnnotator software in the BEAST package with the parameter of Burnin (as states) of 105. Then, the tree generated was edited using FigTree program in the BEAST package. Finally, an outgroup of *Escherichia coli* 16S RNA gene was used as the root tree.

### 2.8. Statistical Analysis

The Cohen Kappa coefficient (k) with 95% confidence interval was used to assess agreement between the commercial ELISA and nested PCR assay (VassarStats program freely available online: http://vassarstats.net/ accessed on 6 April 2023). The strength of agreement is graded as poor when <0.20; fair between 0.21 and 0.40; moderate between 0.41 and 0.60; good from 0.61 to 0.80; or very good from 0.81 to 1 [31].

## 3. Results

In the present study, blood samples were collected in different geographic regions of Mexico from 111 purposively selected dogs with suspected canine monocytic ehrlichiosis. All samples were tested by a commercial ELISA and nPCR assay for the detection of *E. canis*. 

### 3.1. Commercial ELISA Test

Of the 111 samples, 95 (85.6%) were positive for *E. canis* antibodies. Forty-one samples corresponded to Guerrero state, of which 73.1% had a positive result (30/41). In the case of the Morelos state, 20 samples were analyzed, with 75% of the dogs positive (15/20). Finally, in samples from the state of Chihuahua, 100% of the dogs tested positive for *E. canis* antibodies (50/50) (Table 2). The sample size among regions varied in numbers based primarily on the purposive design type of study and were distributed that way because not all owners of sick dogs agreed on participating in the study. 

### 3.2. Nested PCR Assay 

Results of the nested PCR assay on dog samples were as follows (Table 2): Guerrero had 41.4% (17/41) positive samples, Morelos had 55% (11/20), and Chihuahua had 14% (7/50).

### 3.3. Results Comparison of Commercial ELISA and nPCR

The concordance rate between commercial ELISA and nPCR results showed that out of 111 samples, 95 were positive for ELISA, while only 35 were positive for the nPCR. Our results suggest poor agreement between commercial ELISA and nPCR (K ≤ 0.20), with an overall concordance of 35.1% (39/111) (Table 3). 

### 3.4. DNA Sequencing

Out of the 35 positive nPCR samples, it was possible to sequence only 16 DNA fragments corresponding to 45.7%. BLASTn analysis of the sequence showed 100% identity to the *E. canis* 16S rRNA gene (GenBank accession number KF972450.1). The sequences were deposited in GenBank with the following accession numbers: Morelos OP268413.1, OP268414.1, OP268415.1, OP268416.1, OP268417.1, OP268418.1, OP268419.1, OP268420.1, OP268421.1, and OP268422.1; Guerrero OP268423.1, OP268424.1, OP268425.1, OP268426.1, and OP268427.1; Chihuahua OP268428.1.

### 3.5. Phylogenetic Analysis

**Figure 1:**
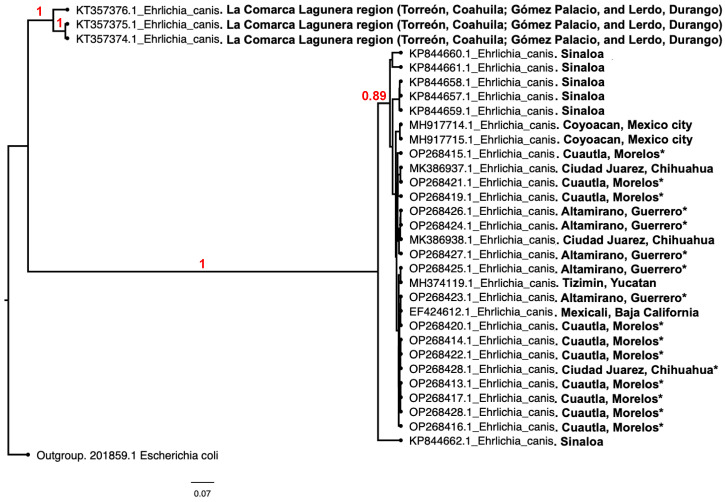
Phylogenetic tree of Bayesian inference of 16S rRNA from Mexican *E. canis* strains. Analysis was conducted with nucleotide sequences of 16S rRNA gene from different strains isolated in different geographic regions of Mexico. The Bayesian phylogenetic tree was estimated using the Bayesian Markov chain Monte Carlo chain, implemented in BEAST. The figure was produced using FigTree (http://tree.bio.ed.ac.uk/software/figtree/, accessed on 13 March 2023). The tree is rooted using *E. coli* as an outgroup. Red numbers in selected nodes are labelled with posterior probability values. Isolates reported in this study are marked with asterisks, the remaining sequences were obtained from GenBank. 16S rRNA gene sequence of *E. coli* was used as an outgroup. Red numbers in selected nodes are labelled with posterior probability values.

The Bayesian topology of the tree was constructed with a total of 31 sequences of 16S rRNA gene of *E. canis* isolated from nine states of Mexico, located in the Nearctic and Neotropical regions (Figure 1). The resulting phylogenetic tree revealed that there is clustering of two groups (Figure 1). The smaller group corresponds to a strain isolated from La Comarca Lagunera, while the largest group corresponds to the rest of the strains from Mexico, including those reported in this study. The conformation of both groups may correspond to geographic trends, a hypothesis that will be discussed.

## 4. Discussion

In the present work, two different diagnostic techniques were used, a commercial ELISA and a nested PCR assay, both type of assays have been reported useful for CME diagnosis [16,17,32,33]. Results showed different infection rates for the three geographical areas studied and for each diagnostic test used; however, markedly higher for ELISA. In the past, the diagnosis of Tropical Canine Pancytopenia relied primarily on the detection of antibodies, and it was usually performed by using indirect fluorescence antibody test (IFAT) and ELISA. However, these tests can mistake the results due to the antigenic cross-reactivity existent among other canine ehrlichial pathogens giving rise to a potential pitfall in the diagnosis of canine ehrlichiosis. Thus, it has been established that during the clinical phase of the disease process, cross-reactivities existent with other ehrlichial and anaplasma species, multiple tick-borne infections, and persistent antibody titers post-treatment must be considered when interpreting *E. canis* diagnostics and, in our case, serological prevalence rates [8,9]. Numerous studies have reported higher prevalence rates for serological compared to molecular tests, given that elicited antibodies after pathogen exposure can persist for months or years, with different antibody titers. It is important to note that a positive serologic test does not necessarily indicate that the pathogen causing disease is present, it only denotes that infection has occurred, unlike molecular tests where a positive result indicates that the infection is active [8,9]. For Cd. Juárez, ELISA results showed 100% serological prevalence (50/50), while in Morelos and Guerrero prevalence rates of 75% (15/20) and 73.1% (30/41), respectively, were determined (Table 2). A previous study in Mexico calculated the seroprevalence of tick-borne diseases [30]. The study divided the country into eight geographical zones, including the three states sampled in this study, and reported 55% prevalence for the northwestern zone (including Chihuahua state), 32% for the southwestern zone (including Guerrero state), and finally, up to 4% for the central south zone (including Morelos state). These areas included more Mexican states than those analyzed in this study. Others have described a 74.3% [33] CME serological prevalence rate for Mexico of, or up to 30% in temperate to cold climate areas [34], implying a wide occurrence of *E. canis* and CME throughout the Mexican Republic. The difference in prevalence reported in this study may be due to the inclusion criteria of dogs studied, and collection time. It is important to note that all dogs included in the present study showed clinical signs compatible with CME and / or previous history of tick infestation. 

Several PCR assay formats have been used to diagnosis CME including real-time PCR (qPCR) and nested PCR (nPCR) [7]. A nPCR targeting the 16S rRNA gene is the most frequently used assay to detect *E. canis* in Mexico. For example, Pat-Nah et al. (2015) reported 36% (18/50) of *E. canis* positive samples by nPCR [35], whereas Ojeda Chi et al. (2019) reported up to 29.46% (72/246) [16]. In the Northern part of the country some studies have also been carried out to detect *E. canis* infection; the presence of *E. canis* DNA was detected in 28% (47/170), 10% (10/100), and 53.6% (104/194) of the samples analyzed by nPCR in Sonora [36], La Comarca Lagunera [15], and Paso del Norte region [18], respectively.

Regarding the phylogenetic analysis of *E. canis* 16S rRNA gene sequences from different geographic regions in Mexico, we found low diversity between different geographic isolates given a high DNA sequence identity. Phylogenetic analysis suggests that the 16S rRNA gene in Mexican *E. canis* from different geographic isolates appears to be highly conserved with few nucleotide differences between them (Figure 1). This low polymorphism suggests a common origin of *E. canis* in Mexico, which is possibly under evolutionary divergence considering there is still no separation between the sequences and their geographical distribution. However, the phylogenetic analyses showed a clade with a common ancestor for all *E. canis* isolates presented. This grouping represents the isolates of Comarca Lagunera [15] that showed a greater divergence than the remaining isolates, and that did not correlate with their geographical distribution. This divergence may be due to the biogeographic isolation of these sequences, since they come from the physiographic region Northern mountain ranges and plains, where components of the geographical space might cause isolation and deter connection with other physiographic regions in Mexico [37]. This phenomenon is similar to that found in Uruguay, where *E. canis* isolates were molecularly characterized and different genotypes correlated to areas of geographical isolation were observed [38]. However, not only the 16S gene was characterized, but also the dsb and groEL genes were included for characterization, increasing the probability of finding differences in the genetic sequences of the gene fragments used.

While the 389 bp fragment amplified from 16S rRNA gene hypervariable region has allowed *E. canis* identification in several studies [15,19,28], it is not possible to conclude that Mexican isolates from La Comarca Lagunera belong to a different genotype, it is suggested to analyze the entire length 16S rRNA gene (approximately 1380 bp) or other genes such as TRP36 [12,19]. 

As mentioned in other studies on *E. canis* phylogenetic characterization, to elucidate the diversity and evolution of strains from different geographical areas and from a medical perspective, a larger genetic polymorphism study of other regions of the *E. canis* genome would be necessary [39]. This is in order to determine whether they substantially affect the virulence of the strain, the preference for the host, and the pathophysiology of the disease.

Diagnosis of CME could be challenging; it is important to consider several aspects, such as clinical history, blood analysis, and the presence of clinical signs compatible with the disease. The use of a serological test might enhance the possibility of diagnosing *E. canis* as the etiological agent. However, as indicated earlier, the recognized serological cross-reactivity of *E. canis* with other rickettsial organisms requires the use of more definitive diagnostic tests for the identification of the infecting organism. A definitive diagnosis of active infection of *E. canis* requires molecular techniques [5], and in chronic cases, samples of target organs might be required for PCR [40]. In a clinical setting, it is recommended that after preliminary CME signs, a combination of serological and PCR tests might increase the probability of an accurate diagnosis [41].

## 5. Conclusions

The 389 bp fragment amplified from 16S rRNA gene hypervariable region has allowed *E. canis* identification and genetic diversity of the *E. canis* 16S rRNA gene in Mexico. Phylogenetic analysis showed that clades are very similar to each other, which suggests the same geographic and evolutive origin. Studies conducted in other countries show that greater genetic diversity in other *E. canis* genes exist, so it is suggested to carry out similar molecular epidemiology studies in Mexico to potentially relate these differences with the degree of disease the various geographically different *E. canis* isolates might cause. It is suggested to analyze the entire length 16S rRNA gene and other genes with higher genetic polymorphism of the *E. canis* genome such as TRP36 to elucidate the diversity and evolution of strains from a larger number of different geographical areas. This could provide insights to determine whether they substantially affect the virulence of the strain, the preference for the host, and the pathophysiology of the disease.

## Figures and Tables

**Table 1:** Sequence of primer sets and protocols used for detection of *Ehrlichia canis* DNA by nested PCR assay.

Pathogen	Oligonucleotide Sequence (5′ to 3′)	PCR Fragment Size (bp)	Reference
*Ehrlichia spp.* (initial PCR)	ECC-AGAACGAACGCTGGCGGCAAGCC ECB-CGTATTACCGCGGCTGCTGGC	478	[24]
*E. canis* (nested PCR)	HE-TATAGGTACCGTCATTATCTTCCCTAT ECA-CAATTATTTATAGCCTCTGGCTATAGGAA	389	[24]

**Table 2:** ELISA and PCR assay results of sampled dogs.

Geographic Region	Total Samples	Commercial ELISA		nPCR	
		Positive Samples	Frequency (%)	Positive Samples	Frequency (%)
Guerrero	41	30	73.1	17	41.4
Morelos	20	15	75	11	55
Chihuahua	50	50	100	7	14
Total	111	95	85.6	35	31.5

**Table 3:** Comparison of the results for dog samples analyzed by commercial ELISA and nPCR assay.

Commercial ELISA	nPCR		
	+	−	Total
+	29	66	95
−	6	10	16
Total	35	76	111

## Data Availability

Data are available from the authors upon reasonable request.
